# Parenting styles and health in mid- and late life: evidence from the China health and retirement longitudinal study

**DOI:** 10.1186/s12877-022-03157-6

**Published:** 2022-05-28

**Authors:** Ruoxi Ding, Ping He

**Affiliations:** China Center for Health Development Studies, Peking University, Beijing, 100191 China

**Keywords:** Parenting style, Parental affection, Parental discipline, Mid- and late life, Health outcome

## Abstract

**Backgrounds:**

The impact of relationships in early childhood may be long-lasting and reaching to mid to late life. Limited studies have investigated the associations between parenting style and different aspects of well-being beyond adolescence. The current study aims to examine the association between parenting styles and multiple dimensions of functioning in mid-and later-life adults.

**Methods:**

We used data from China Health and Retirement Longitudinal Study (CHARLS). Generalized Estimating Equation (GEE) was applied to examine the association between retrospective parenting styles/behaviors in childhood and health outcome.

**Results:**

Compared with authoritative style, authoritarian style predicted worse self-rated health (coefficient = − 0.13, *P* < 0.001), cognitive function (− 0.23, *P* < 0.05) and depressive symptom (0.87, *P* < 0.001). Paternal affection was associated with more health outcome in mid- and late life than maternal affection. Only paternal affection was a significant predictor of mid- and late life health among male adults, while both paternal and maternal affection were strong predictors among female adults. Authoritative style was associated more positive health outcomes in mid- and late life among adults with literate parents than those with illiterate parents.

**Conclusion:**

This study provides evidence for the link between parenting behaviors in early life stage and physical and psychological functioning in mid- to late adulthood. Authoritative style, and the memory of parental affection, particularly from father and educated parents, could have long-lasting positive influence on children’s physical and mental well-being, which further support the life-course perspective on human development.

**Supplementary Information:**

The online version contains supplementary material available at 10.1186/s12877-022-03157-6.

## Introduction

As the immediate social surrounding for an individual, family is the critical source of social support that influences future development and well-being [1]. Close and positive relationship between family members has been hypothesized as an essential element for maintaining satisfactory health trajectory [2] and promoting flourishing [3]. Specifically, parenting practices in early time may exert paramount effect across one’s life span because it can potentially facilitate or hinder children’s overall adaption functioning [4]. For instance, positive parenting practice often provides children with social integration and emotional security, which function as psychosocial resources in later life that help increasing resilience and further promoting well-being in multiple aspects [5]. Conversely, children under negative parenting would be more vulnerable to emotional dysregulation and psychological distress, as well as risky health behaviors and further suffer from long-term health consequence [6, 7].

Primarily two domains of parenting behaviors were considered critical to individual’s development by most theories of family socialization: parental affection and parental discipline [8]. Adequate parental affection predicts a strong sense of security and self-worth and thereby reduces children’s risk of experiencing anxiety, aggression, and low self-esteem. Parental discipline refers to the rules and guidelines that parents set and enforce, which teaches children about group and social standards for behaviors to improve self-control and shape responsible conformity [8, 9]. Based on levels of parental affection and parental discipline, four types of parenting styles have been identified on a regular basis: the authoritative (high in both affection and discipline), authoritarian (low in affection and high in discipline), indulgent (high in affection and low in discipline) and uninvolved style (low in both affection and discipline) [10, 11]. Most evidence suggested the authoritative style, as the advantaged parenting practice, was associated with better physical health and mental well-being in adolescence or young adulthood [12–15].

The impact of relationships in early childhood may be long-lasting and reaching to mid to late life. Specifically, from the life-course perspective [16], the influence of parenting practice does not cease after adolescence but continues to operate across the entire life span. However, very limited studies have investigated the associations between parenting style and different aspects of well-being beyond adolescence or young adulthood. And less is known from the current studies considering the relative magnitudes of the contributions of maternal and paternal affection and discipline on physical and mental health in mid and later life.

Research on children and adolescents indicated that the effect of parenting practice may differ somewhat based on children’s gender and family socioeconomic status [17–19]. In most cultures, parents often differ in their expectation for sons versus daughters, as well as the manners socializing them. And boys and girls may also perceive parenting behaviors differently and further respond in different ways, particularly during adolescence [17, 18]. Besides, family socioeconomic conditions, parental education in particular, may shape their personal values and further impact on parenting practice. Parents with limited educational backgrounds are more likely to neglect their children’s higher needs like self-fulfillment [19], which can impede reaching their potentials and affect well-being in adulthood.

As such, the current study aims to examine the association between parenting styles and multiple dimensions of functioning in mid-and later-life adults, as well as the contributions of maternal and paternal affection and discipline. We hypothesized that memories of authoritative parenting behavior are associated with better health outcomes (self-rated health, cognitive function and depressive symptoms) in mid- and late life comparing to the memories of authoritarian, indulgent and uninvolved parenting styles. To further illustrate the parenting-health linkage, we also investigated whether parental affection and discipline are independently correlated with the late-life health outcome, as well as the heterogeneity in above associations between males and females, and those with literate parents and illiterate parents.

## Methods

### Participants

The analyses used data from China Health and Retirement Longitudinal Study (CHARLS). CHARLS is a biennial survey that designed to investigate on a nationally representative sample of Chinese residents in mainland China aged 45 years and older. It employed multi-stage stratified sampling and covered 28 provinces and 150 counties. The survey sample included more than 14,000 households and 25,000 individuals. The first, second and third national survey were conducted in 2011, 2013 and 2015, respectively. Respondents’ demographic information, health status and functioning, health care and insurance, socio-economic status, etc. were collected in all these biennial core survey waves. Additionally, in 2014, a special life history survey was conducted to collect information about history of residence, relocation, education, health and health care history of all longitudinal samples. The overall response rate of 2011, 2013, 2014 and 2015 survey was 80.5, 88, 86 and 87%, respectively. Detailed descriptions about the design and sampling procedure of this survey were provided elsewhere [20].

We matched the individuals of 2011, 2013, 2014 and 2015 survey based on their individual ID to obtain their information of childhood experience (parenting behaviors, parental mental illness, economic disadvantages, and health status), sociodemographic data and information on health status. Overall, 17,708, 18,612 and 21,097 samples were collected in 2011, 2013 and 2015 surveys, respectively. We excluded 386 participants with both parents died during childhood, and 4974 participants with missing data on covariates (age, sex and urban-rural residence, education attainment, marital status, financial status, childhood socioeconomic disadvantages, childhood socioeconomic disadvantages, parental mental illness and childhood health status), totally 12,493 individuals were included in final analysis (The mean score of outcome variables among included and excluded participants were provided in appendix Table 1).

### Measurement

#### Outcomes variables

##### Self-rated health

The self-rated health status is based on a 5-point scale from “1= very poor” to “5= very good”.

##### Cognitive function

In CHARLs, cognitive function was assessed based on mainly two domains: mental intactness and episodic memory. Mental intactness was tested by a battery of cognitive tasks including naming today’s date (months, day, year), and recalling the day of the week and the present season, subtracting 7 from 100 up to 5 times, and redrawing a picture shown by the interviewer. Episodic memory was assessed by the immediate and delayed word recall test. The variable of cognitive function (global cognitive function) is the aggregate score of these two domains, which is ranging from 0 to 21, with a higher score suggesting a better cognitive function. The reliability of the cognition scale in CHARLs has been proved to be excellent by previous research [21].

##### Depressive symptoms

The variable of depressive symptoms was employed based on the aggregated score of the Center for Epidemiologic Studies Depression Scale (CES-D-10) used in CHARLs, which ranges from 0 to 30, with a higher score suggesting more depressive symptoms during the previous week. The scale has indicated adequate reliability and validity for the community-dwelling older population in China [22]. The CES-D-10 scale includes 10 items asking about respondents’ experience during the past week: feeling bothered, having trouble in concentrating, feeling depressed, feeling as though everything was effortful, feeling hopeful, feeling fearful, having restless sleep, feeling happy, feeling lonely, and having difficulty in getting going.

#### Independent variables

##### Maternal/paternal affection and discipline

In this study, the childhood period was defined as before 17 years old in CHARLS Life History Survey. Based on the parenting dimensions proposed by previous literature [10], studies on parenting behaviors [11, 23] and the question in CHARLS life history questionnaire, we constructed the parental affection and discipline scales separately for mother and father. Maternal affection scale included three items: “How would you rate your relationship with your female guardian when you were growing up” (rated on a 5-point scale ranging from 1(excellent) to 5 (poor)), “How much love and affection did your female guardian give you while you were growing up?” (rated on a 4-point scale ranging from 1(often) to 4 (never)) and “How much effort did your female guardian put into watching over you?” (rated on a 4-point scale ranging from 1(often) to 4 (never)). To create continuity among items, they were recoded, standardized and averaged such that a higher score indicates a higher level of maternal affection (The Cronbach’s alpha for these three items was 0.631). Paternal affection was assessed based on a single item: “How would you rate your relationship with your male guardian when you were growing up” (rated on a 5-point scale ranging from 1(excellent) to 5 (poor)), which was also recoded to be consistent with maternal affection. Maternal and paternal discipline were assessed based on a single item: “How strict was your female (male) guardian with her (his) rules for you? (rated on a 5-point scale ranging from 1(very strict) to 4 (not at all strict)).

##### Parenting styles

Next, based on the categorization approaches used in the parenting and child development literature [11, 24], we calculated the mean across the maternal and paternal affection and discipline as the two parental dimension scale. Then we used the median split on the parental affection and discipline to establish low and high levels of affection and discipline. Finally, four parenting styles were developed: Authoritative (high affection and high discipline), authoritarian (low affection and high discipline), indulgent (high affection and low discipline) and uninvolved (low affection and low discipline).

#### Covariates

Potential covariates were identified and adjusted based on previous research on similar topics [11, 23, 25] and life-course research using CHARLs survey [26–28]: age, as continuous variable, sex (1 = male, 0 = female), survey year (2011, 2013, 2015), residence (1 = rural, 0 = urban), education attainment, as a standardized score based on the years of education reported by respondents and marital status, as categorized variable (1 = married/partnered, 2 = separated/divorced/widowed, 3 = single). We also include financial status. Because of a large number of missing values in self-reported household income or wealth in CHARLS, we used the standardized score of annual household expenditure per capita as the alternative variable to reflect financial status. It has been suggested by evidence that household expenditure could provide a more reliable and valid assessment of living standards and financial status in developing nations [29].

Additionally, in the light of the findings from previous studies on similar topics [11, 23, 25] and studies based on CHARLs survey exploring potential childhood risk factors for late life health outcomes [30–32], our analyses also adjusted for potential important confounders in childhood: 1. Childhood socioeconomic disadvantage, which is a proxy for childhood socioeconomic status. In this study, childhood socioeconomic disadvantage was an aggregated count (0–4) of four dichotomous indicators that reported in Life history survey: 1) Both parents were illiterate (1 = yes or 0 = no); 2) Hunger experience (1 = yes or 0 = no); 3) Flee from famine (1 = yes or 0 = no), 4) Worse economic situation than neighbors (1 = yes or 0 = no). 2. Parental mental illness in childhood (One or both of respondent’s parents feel nervous, anxious, panicky, sadness, depression or low energy in a good part of the time or most of the time, or his/her parents have abnormality of mind in his/her childhood, 1 = yes, 0 = no) and 3. childhood serious physical illness (Respondent ever confined to bed or home/ hospitalized for a month or more, or hospitalized more than three times within a 12-month period before 15 years old, 1 = yes, 0 = no).

### Statistical analysis

Descriptive statistics were conducted to report the distribution in demographic characteristics, the proportion of each type of parenting styles, the mean score of parental affection and discipline and the mean score of health outcome in mid- and late life of our sample. Generalized Estimating Equation (GEE) was applied to examine the association between parenting styles and health outcome (self-rated health, global cognitive function and depressive symptoms) (Model 1–4). GEE was recommended as the efficient model for the analysis of the longitudinal dataset with repeated measures. The main advantage of GEE resides in the unbiased estimation of population-averaged regression coefficients despite possible misspecification of the correlation structure [33]. To further explore the relative magnitudes of the contributions of different parenting dimensions, we conducted GEE analysis on the association between parental affection and discipline and health outcome in mid- and late life (Model 5–8). Additionally, subsample analyses were also applied to investigate the heterogeneity in above associations between male and female, and respondents with literate parents and those with illiterate parents. All the analyses were conducted with Stata 15.0.

## Results

### Sample characteristics

Table 1 presents demographics, distribution of parenting styles and behaviors, and mean score of health outcome of the study sample. The mean age of 12,493 adults was 57.14 years old, 48.94% of respondents were male, 39.69% lived in urban areas, and 91.73% were married/partnered. Overall, 2237 (17.91%), 1486(11.89%), 4125(33.02) and 4645(37.18) respondents reported authoritative, authoritarian, indulgent and uninvolved style in childhood, respectively. The mean score for maternal affection, paternal affection, maternal discipline, and paternal discipline were 4.03, 3.73, 2.71 and 2.70, respectively. The mean score for self-rated health was 2.24, and the mean score of global cognitive function and CES-D-10 were 11.58 and 7.70. The characteristics of male and female sample were also provided in Table 1.

**Table 1:** Sample characteristics

	**Mean (SD)/N(%)**		
**Demographic characteristics**	**All** **(*N* = 12,493)**	**Male** **(*N* = 6114)**	**Female** **(*N* = 6379)**
**Age**	57.70 (9.25)	58.73 (9.11)	56.68 (9.26)
**Residence**			
Urban	4959 (39.69)	2355 (38.52)	2604 (40.82)
Rural	7534 (60.31)	3759 (61.48)	3775 (59.18)
**Marital status**			
married/partnered	11,460 (91.73)	5689 (93.05)	5771 (90.47)
separated/divorced/widowed	957 (7.66)	355 (5.81)	602 (9.44)
single	76 (0.61)	70 (1.15)	6 (0.09)
**Parenting styles**			
Authoritative	2237 (17.91)	1161 (18.99)	1076 (16.87)
Authoritarian	1486 (11.89)	797 (13.04)	689 (10.80)
Indulgent	4125 (33.02)	1883 (30.08)	2242 (35.15)
Uninvolved	4645 (37.18)	2273 (37.18)	2372 (37.18)
**Maternal Affection (1–4.34)**	3.48 (0.76)	3.49 (0.75)	3.47 (0.77)
**Paternal Affection (1–5)**	3.73 (1.11)	3.68 (1.11)	3.78 (1.09)
**Maternal discipline (1–4)**	2.71 (1.07)	2.76 (1.04)	2.67 (1.11)
**Paternal discipline (1–4)**	2.70 (1.10)	2.79 (0.97)	2.61 (1.02)
**Parental illiteracy**			
Both parents were literate	6564 (52.54)	3245 (53.07)	3319 (52.03)
One of parents was illiterate	5929 (47.46)	2869 (46.93)	3060 (47.97)
**Self-rated health (1–5)**	2.24 (0.95)	2.32 (0.95)	2.17 (0.93)
**Global cognitive function (0–21)**	11.58 (3.86)	12.19 (3.47)	10.99 (4.13)
**CES-D-10 score (0–30)**	7.70 (6.08)	6.80 (5.56)	8.56 (6.41)

*SD* Standard deviation, *CES-D-10* 10-item Centre for Epidemiological Studies Depression Scale

Table 2 displays the mean scores of health outcomes among participants who reported different parenting styles. Participants reported authoritative style have better self-rated health, better cognitive function and fewer depressive symptoms compared with participants reported authoritarian or uninvolved parenting styles. Similar patterns were observed for male and female sample.

**Table 2:** Mid- and late life health outcome by parenting styles (Mean (SD))

	**Self-rated Health**	**Cognitive function**	**CES-D-10 score**
**Parenting styles**	**All**		
Authoritative	2.32 (0.99)	11.67 (3.74)	7.49 (6.11)
Authoritarian	2.18 (0.93)	11.38 (3.76)	8.54 (6.23)
Indulgent	2.31 (0.97)	11.78 (3.86)	7.26 (6.03)
Uninvolved	2.17 (0.89)	11.42 (3.95)	7.92 (6.02)
*P* value	< 0.001	< 0.001	< 0.001
	**Male**		
Authoritative	2.40 (1.00)	12.32 (3.38)	6.63 (5.63)
Authoritarian	2.28 (0.96)	11.88 (3.48)	7.58 (5.86)
Indulgent	2.38 (0.97)	12.43 (3.39)	6.27 (5.43)
Uninvolved	2.24 (0.90)	12.03 (3.57)	7.04 (5.49)
P value	< 0.001	< 0.001	< 0.001
	**Female**		
Authoritative	2.23 (0.98)	*10.94 (3.98)*	8.39 (6.46)
Authoritarian	2.06 (0.89)	*10.78 (3.99)*	9.65 (6.47)
Indulgent	2.25 (0.97)	11.24 (4.14)	8.09 (6.37)
Uninvolved	2.09 (0.88)	10.82 (4.20)	8.76 (6.37)
P value	< 0.001	0.004	< 0.001

*SD* Standard deviation, *CES-D-10* 10-item Centre for Epidemiological Studies Depression Scale

### Association between parenting style/parenting behaviors and health outcome in mid- and late life

Table 3 presents the association of parenting style and parenting behaviors with mid- and late life health outcome. Overall, estimates in Model 1 to Model 4 suggest that after adjusting for covariates and potential confounding variables, parenting styles was associated with all four types of health outcome. Compared with authoritative style, authoritarian style was associated with lower level of self-rated health (coefficient = − 0.13, *P* < 0.001), a lower score of cognitive function (− 0.23, *P* < 0.05) and more depressive symptoms (0.87, *P* < 0.001), and uninvolved style was associated with a lower level of self-rated health (− 0.13, *P* < 0.001) and more depressive symptoms (0.30, *P* < 0.05). Additionally, indulgent style was associated with a higher score of global cognitive function (0.25, *P* < 0.001) and more depressive symptoms(− 0.34, *P* < 0.05) than authoritative style.

**Table 3:** Generalized Estimating Equation on the association between parenting styles (behaviors) and health outcome in mid- and late life

	**Model 1**^**a**^	**Model 2**^**a**^	**Model 3**^**a**^
	**Self-rated Health**	**Cognitive function**	**CES-D-10 score**
**Parenting styles**			
Authoritative	Reference	Reference	Reference
Authoritarian	− 0.13^***^	−0.23^*^	0.87^***^
Indulgent	0.02	0.25^***^	− 0.34^*^
Uninvolved	−0.12^***^	− 0.01	0.30^*^
	**Model 4**^**a**^	**Model 5**^**a**^	**Model 6**^**a**^
	**Self-rated Health**	**Cognitive function**	**CES-D-10 score**
Maternal affection	0.04^***^	0.05	−0.15^*^
Maternal discipline	−0.01	− 0.03	0.15^**^
Paternal affection	0.05^***^	0.10^**^	−0.29^***^
Paternal discipline	−0.02^*^	−0.02	0.02

^a^Adjusted for age, gender, survey year, residence, marital status, education, financial status, childhood socioeconomic disadvantages, childhood health status and parental mental illness

***P < 0.001; **P < 0.01; *P < 0.05

*CES-D-10* 10-item Centre for Epidemiological Studies Depression Scale

And estimates in Model 5 to Model 8 suggest that parenting behaviors was associated with self-rated health, global cognitive function and depressive symptoms. Maternal and paternal affection were associated a higher level of self-rated health, a higher score of cognitive function and fewer depressive symptoms. And maternal discipline was associated with more depressive symptoms (0.15, *P* < 0.01), while paternal discipline was associated with a lower level of self-rated health (− 0.02, *P* < 0.05).

### Male and female subsample analyses

Table 4 and Table 5 presents male and female subsample analyses on the association of parenting style and parenting behaviors with mid- and late life health outcome. GEE models suggested that parenting style was associated with self-rated health, cognitive function and depressive symptoms among male respondents. Compared with authoritative style, authoritarian style was associated with a lower level of self-rated health(− 0.13, *P* < 0.01), and more depressive symptoms (0.79, *P* < 0.001), uninvolved style was associated with a lower level of self-rated health(− 0.12, *P* < 0.01) and indulgent style was associated with a higher score of cognitive function and fewer depressive symptoms (− 0.37, *P* < 0.05). Analyses on parenting behaviors suggests that only paternal affection was associated with a higher level of self-rated health (0.06, *P* < 0.001), and fewer depressive symptoms (− 0.35, *P* < 0.001), and maternal discipline was associated with a lower score of cognitive function (− 0.10, *P* < 0.05).

**Table 4:** Generalized Estimating Equation on the association between parenting styles (behaviors) and health outcome in mid- and late life (male subsample)

	**Model 1**^**a**^	**Model 2**^**a**^	**Model 3**^**a**^
	**Self-rated Health**	**Cognitive function**	**CES-D-10 score**
**Parenting styles**			
Authoritative	Reference	Reference	Reference
Authoritarian	−0.13^**^	− 0.19	0.79^***^
Indulgent	0.01	0.20^*^	−0.37^*^
Uninvolved	−0.12^**^	−0.05	0.29
	**Model 4**^**a**^	**Model 5**^**a**^	**Model 6**^**a**^
	**Self-rated Health**	**Cognitive function**	**CES-D-10 score**
**Parenting behaviors**			
Maternal affection	0.02	0.06	−0.06
Maternal discipline	−0.01	− 0.10^*^	0.09
Paternal affection	0.06^***^	0.06	−0.35^***^
Paternal discipline	−0.01	0.04	0.05

^a^Adjusted for age, survey year, residence, marital status, education, financial status, childhood socioeconomic disadvantages, childhood health status and parental mental illness

^***^P < 0.001; ^**^P < 0.01; ^*^P < 0.05

*CES-D-10* 10-item Centre for Epidemiological Studies Depression Scale

**Table 5:** Generalized Estimating Equation on the association between parenting styles (behaviors) and health outcome in mid- and late life (female subsample)

	**Model 1**^**a**^	**Model 2**^**a**^	**Model 3**^**a**^
	**Self-rated Health**	**Cognitive function**	**CES-D-10 score**
**Parenting styles**			
Authoritative	Reference	Reference	Reference
Authoritarian	−0.13^***^	− 0.32^*^	0.96^**^
Indulgent	0.04	0.30^**^	−0.31
Uninvolved	−0.12^***^	0.02	0.32
	**Model 4**^**a**^	**Model 5**^**a**^	**Model 6**^**a**^
	**Self-rated Health**	**Cognitive function**	**CES-D-10 score**
**Parenting behaviors**			
Maternal affection	0.05^**^	0.05	−0.23^*^
Maternal discipline	−0.01	0.01	0.20^*^
Paternal affection	0.03^**^	0.14^**^	−0.23^**^
Paternal discipline	−0.03^*^	−0.07	0.01

^a^ Adjusted for age, survey year, residence, marital status, education, financial status, childhood socioeconomic disadvantages, childhood health status and parental mental illness

^***^P < 0.001; ^**^P < 0.01; ^*^P < 0.05

*CES-D-10* 10-item Centre for Epidemiological Studies Depression Scale

For female subsample, parenting styles were associated with all four types of health outcome. Compared with authoritative style, authoritarian style was associated with a lower level of self-rated health (− 0.13, *P* < 0.001), a lower score of cognitive function (− 0.32, *P* < 0.05) and more depressive symptoms (0.96, *P* < 0.01), uninvolved style was associated with a lower level of self-rated health (− 0.12, *P* < 0.001), while indulgent style was associated with a higher score of cognitive function (0.30, *P* < 0.01). Analyses on parenting behaviors suggest that both maternal and paternal affection were associated with higher level of self-rated health and fewer depressive symptoms. Maternal discipline was associated with more depressive symptoms and paternal discipline was associated with a lower level of self-rated health.

### Illiterate and literate parents subsample analyses

Table 6 and Table 7 presents analyses on the association of parenting style and parenting behaviors with mid- and late life health outcome among adults with illiterate parents and those with literate parents. GEE models suggested that, compared with authoritative style, authoritarian style was associated with a lower level of self-rated health (− 0.15, *P* < 0.001) and more depressive symptoms (0.76, *P* < 0.01), uninvolved style was associated with a lower level of self-rated health (− 0.14, *P* < 0.001), while indulgent style was associated with a higher score of cognitive function (0.30, *P* < 0.01) and fewer depressive symptoms (− 0.38, *P* < 0.05) in adults with illiterate parents. Analyses on parenting behaviors showed that maternal affection was only associated with a lower level of self-rated health, and paternal affection was a predictor for self-rated health and depressive symptoms. Additionally, maternal discipline was associated with more depressive symptoms and paternal discipline was associated with a lower level of self-rated health.

**Table 6:** Generalized estimating equation on the association between parenting styles (behaviors) and health outcome in mid- and late life (subsample from families with at least one illiterate parent)

	**Model 1**^**a**^	**Model 2**^**a**^	**Model 3**^**a**^
	**Self-rated Health**	**Cognitive function**	**CES-D-10 score**
**Parenting styles**			
Authoritative	Reference	Reference	Reference
Authoritarian	−0.15^***^	−0.23	0.76^**^
Indulgent	0.01	0.30^**^	−0.38^*^
Uninvolved	−0.14^***^	0.01	0.24
	**Model 4**^**a**^	**Model 5**^**a**^	**Model 6**^**a**^
	**Self-rated Health**	**Cognitive function**	**CES-D-10 score**
**Parenting behaviors**			
Maternal affection	0.04^**^	0.07	−0.11
Maternal discipline	0.01	−0.03	0.14^*^
Paternal affection	0.05^***^	0.08	−0.27^***^
Paternal discipline	−0.03^*^	−0.03	0.06

^a^ Adjusted for age, gender, survey year, residence, marital status, education, financial status, childhood socioeconomic disadvantages, childhood health status and parental mental illnesss

^***^P < 0.001; ^**^P < 0.01; ^*^P < 0.05

CES-D-10; 10 item Centre for Epidemiological Studies Depression Scale

**Table 7:** Generalized estimating equation on the association between parenting styles (behaviors) and health outcome in mid- and late life (subsample from families with literate parents)

	**Model 1**^**a**^	**Model 2**^**a**^	**Model 3**^**a**^
	**Self-rated Health**	**Cognitive function**	**CES-D-10 score**
**Parenting styles**			
Authoritative	Reference	Reference	Reference
Authoritarian	−0.11^**^	−0.31^*^	0.90^**^
Indulgent	0.01	0.23^*^	−0.15
Uninvolved	−0.09^**^	−0.03	0.35^*^
	**Model 4**^**a**^	**Model 5**^**a**^	**Model 6**^**a**^
	**Self-rated Health**	**Cognitive function**	**CES-D-10 score**
**Parenting behaviors**			
Maternal affection	0.04^*^	0.04	−0.20^*^
Maternal discipline	−0.01	− 0.04	0.16
Paternal affection	0.05^***^	0.12^***^	−0.28^**^
Paternal discipline	−0.01	−0.03	− 0.05

^a^ Adjusted for age, gender, survey year, residence, marital status, education, financial status, childhood socioeconomic disadvantages, childhood health status and parental mental illness

^***^P < 0.001; ^**^P < 0.01; ^*^Ps < 0.05

CES-D-10; 10 item Centre for Epidemiological Studies Depression Scale

In adults with literate parents, parenting style was predictor for all four types of health outcome in mid- and late life. Compared to authoritarian style, authoritative was associated with a lower level of self-rated health(− 0.11, *P* < 0.01), a lower score of cognitive function(− 0.31, *P* < 0.05) and more depressive symptoms (0.90, *P* < 0.01), uninvolved style was associated with a lower level of self-rated health (− 0.09, *P* < 0.01) and more depressive symptoms (0.35, *P* < 0.05), and indulgent style was associated with a higher score of cognitive function (0.23, *P* < 0.05). Analyses on parenting behaviors suggest that maternal affection was a predictor for self-rated health and depressive symptoms, paternal affection was a predictor for self-rated health, cognitive function and depressive symptoms. No significant association was observed between maternal/paternal discipline and any type of health outcome in adults with literate parents.

## Discussion

Based on nationally representative data, this study found significant associations between established parenting styles in childhood and multiple dimensions of health status in midlife and beyond, with authoritative style associated with better physical and mental health status compared to authoritarian or uninvolved styles, and parental affection plays the key role in such associations. Additionally, there were also heterogeneity of gender and parental education. Instead of correlating to both paternal and maternal affection among female sample, only paternal affection was associated with mid- and late life health among male sample. And authoritative styles were associated with more positive health outcomes among those with educated parents than those with illiterate parents.

First, our results indicated that authoritative parenting style in childhood predicted more positive health outcomes in mid- and late life than authoritarian or uninvolved styles, which supported the main part of our hypothesis. And further analysis showed that parental affection was associated with better self-rated health and cognitive function and fewer depressive symptoms, while higher parental discipline was associated with worse self-rated health and mental health status. These findings are consistent with previous studies on child development and adult health [14, 15, 34, 35], and further support the principles of the life course perspective by suggesting that the influence of parenting behaviors could extend into late life. Positive interactions with parents during childhood always linked to experience of more supportive social relationships, which foster well-being and contribute to better physical and mental health [13]. Besides, warm memory of parental affection in childhood could also provide benefit that help reducing stress and promote positive health behaviors [36, 37]. On the other side, negative discipline and excessive control from parents may increase anxiety and depressive symptoms and affect the following health trajectory in adulthood [34, 38]. Furthermore, Chinese traditional culture emphasizes parental discipline and the obedience of children. Parents of our sample with high level of discipline, were more likely to set up strict rules and did not allow their children to have privacy or to oppose family decisions [39]. These harsh characteristics may exacerbate children’s vulnerability to stress and thus increase the risk of unhealthy behavior and disrupted neurobiological functioning and contribute to the suboptimal health status in adulthood.

Second, we found that, compared to maternal affection, paternal affection was positively associated with more health outcome in mid- and late life. This finding is consistent with a previous study on Chinese adolescents [40], which suggested that paternal attachment had a stronger effect on children’s depressive symptoms compared to maternal attachment. It has been pointed out that fathers’ involvement in rearing children could promote the children’s development of social capability and autonomy, which may prevent future adverse experience and subsequent negative health consequence [41]. Moreover, the greater effect of paternal parenting behaviors might be the unique characteristics in China. Though in most cultures, mother take the primary responsibility to take care of children, in Chinese traditional culture, father always have the highest authority in the family [40], so that their affection could influence important decisions in the family and bring substantial benefits for children’s development.

We observed no significant advantage in mid- and late life health outcome for adults reported authoritative parents compared to those who reported indulgent parents. And the health outcome was even better for adults with indulgent parenting styles in the case of cognitive function and depressive symptoms. This is different from our hypothesis and inconsistent to previous research on children and adolescents, which suggests that indulgent style was associated with fewer positive outcomes compared to authoritative style [42]. However, our result was similar to one study focusing on health in mid- and late adulthood from United States [11]. Integrated with the analysis on parental affection and discipline, the possible explanation is that, compared with parental discipline, parental affection was a positive predictor of more health outcome in late life. Even under the circumstances of low discipline, the high level of parental affection could provide crucial emotional support, and function as a buffer for potential negative consequence in adulthood. High-supportive and low-control parents may also have greater bonding and intimacy with children and benefit their mental health in the long run.

Additionally, the gender-specific results showed that only paternal affection was significant predictor of mid- and late life health among male adults. However, for female in the study, both paternal and maternal affection were strong predictors. Similar findings were also reported by one study on adolescents, which suggested that attachment to father was associated with social adjustment and self-efficacy among male adolescents while such association were observed for both parental attachments among female adolescents [43]. It has been suggested that daughter was more susceptible to maternal parenting behaviors compared to son [44]. And besides the above mentioned greater authority of father in Chinese traditional families, the heterogeneity may also be explained by findings from previous literature, which showed that parental care in the same-sex dyads was positively correlated to affective empathy and function as a protective factor against physical and mental health decline [35, 44].

Similarly, there are also difference in the parenting-health linkage between adults with illiterate parents and those with educated parents. Authoritative style was associated with more positive health outcomes in mid- and late life among adults with educated parents than those with illiterate parents. And further analysis provides some explanation: for adults from illiterate family, parental affection was associated with fewer positive outcomes, but parental discipline was associated with negative health consequences, which result in less pronounced benefits of authoritative styles than that in literate families. Previous research on offspring’s locus of control and achievements indicated that parents with higher education always show more efficient parenting behaviors [25]. They identify the connection between children behavior and resultant outcome more clearly by delivering rewards and punishments [45]. And it has also been revealed that the negative emotion of parents with limited educational background had more harmful effect for children’s development compared to that of parents with a higher level of socioeconomic backgrounds [46]. Such evidence may provide thoughts for the underlying mechanism for the less benefits of parental affection and the adverse impact of parental discipline in illiterate families.

Certain limitations in the current study are worth acknowledging. First, due to the retrospective design, the reliability and validity of the parenting behavior reported were compromised. However, as Henry et al. [47] argued, retrospective measure may represent individual’s current perception, which was of interest for social studies and contribute valuable information. Nevertheless, further study is warranted to rule out the possibility that participants’ current cognitive function could impact on memories of childhood experience. Second, although a wide range of childhood characteristics were included, our analysis failed to adjust for other potential residual confounding factors such as parental physical health status, of which information was not available in the survey. This may also result in potential bias for our findings. Third, in CHARLs life history survey, the difference in the measurement of paternal affection (one item) and maternal affection (three items) may also lead to recall bias. Fourth, annual household expenditure was employed as financial status instead of the household income in the analyses, because of which our estimation may be further compromised. Additionally, we excluded a large number of subjects with missing values in independent or outcome variables. This may reduce the statistical power and induce bias to out estimation. Finally, due to the sampling strategy of CHARLs survey, our study failed to include institutionalized adults, which may have reduced the accuracy of estimation on potential significant associations.

## Conclusion

This study provides evidence for the link between parenting behaviors in early life stage and physical and psychological functioning in mid- to late adulthood from the perspective of a non-Western context. Authoritative style, and the memory of parental affection, particularly from father and educated parents, could have long-lasting positive influence on children’s physical and mental well-being, which further support the life-course perspective on human development. Our findings strengthen the evidence for a public health focus on improving parenting and reinforce the importance of targeting parenting as prevention and intervention programs to promote population health and well-being, as well as the program evaluation with respect to their impact on developmental trajectories, especially for children with disadvantaged family backgrounds.

## Supplementary Information

**Additional file 1.**

## Data Availability

The datasets generated analysed during the current study are available at http://charls.pku.edu.cn
