# Supplementary material for: Parenting styles and health in mid- and late life: evidence from the China health and retirement longitudinal study
Source: BMC Geriatr. 2022 May 28;22:463. doi: 10.1186/s12877-022-03157-6 (PMC9145460; doi:10.1186/s12877-022-03157-6)
Supplement: Supplementary file 1 — Additional file 1. [file 12877_2022_3157_MOESM1_ESM.docx]

**Appendix table 1. Mean score of self-rated health, number of chronic conditions, global cognitive score and CES-D-10 score among included and excluded participants.**

|  | 2011 | | |
| --- | --- | --- | --- |
|  | **Self-rated health** | **Global cognitive score** | **CESD-10 score** |
| Included participants | 2.18 | 11.45 | 8.19 |
| Excluded participants | 2.14 | 10.89 | 8.22 |
| P value | 0.099 | <0.001 | 0.21 |
|  | **2013** | | |
| Included participants | 2.21 | 11.52 | 7.69 |
| Excluded participants | 2.19 | 10.93 | 7.74 |
| P value | 0.290 | <0.001 | 0.070 |
|  | **2015** | | |
| Included participants | 2.24 | 11.45 | 7.61 |
| Excluded participants | 2.20 | 10.76 | 7.65 |
| P value | 0.180 | <0.001 | 0.090 |

CES-D-10; 10-item Centre for Epidemiological Studies Depression Scale.

**Appendix table2. Generalized Estimating Equation on the association between parenting styles (behaviors) and health outcome in mid- and late life**

|  | **Model 1^1^** | **Model 2^1^** | **Model 3^1^** |
| --- | --- | --- | --- |
|  | **Self-rated Health** | **Cognitive function** | **CES-D-10 score** |
| **Parenting styles** |  |  |  |
| Authoritative | Reference | Reference | Reference |
| Authoritarian | -0.13^***^ | -0.23^*^ | 0.87^***^ |
| Indulgent | 0.02 | 0.25^***^ | -0.34^*^ |
| Uninvolved | -0.12^***^ | -0.01 | 0.30^*^ |
| Age | -0.01^***^ | -0.07^***^ | 0.04^***^ |
| Male (ref=female) | 0.17^***^ | 0.60^***^ | -1.67^***^ |
| Survey year (ref=2011) |  |  |  |
| 2013 | 0.04^**^ | 0.03 | -0.43^***^ |
| 2015 | 0.05^***^ | -0.16^***^ | -0.35^***^ |
| Rural residence (ref=urban residence) | -0.10^***^ | -0.86^***^ | 1.12 |
| Marital status |  |  |  |
| separated/divorced/widowed | -0.09 | -0.06^**^ | 1.94^***^ |
| single | -0.29^**^ | -1.22^***^ | 2.68^***^ |
| Education attainment score | 0.03^***^ | 1.80^***^ | -0.65^***^ |
| Financial status | 0.01^*^ | 0.12^***^ | -0.01 |
| Childhood socioeconomic disadvantages | -0.06^***^ | -0.17^***^ | 0.50^***^ |
| Childhood serious physical illness (ref=none) | -0.18^***^ | -0.24^*^ | 1.23^***^ |
| Parental mental illness (ref=none) | -0.13^***^ | -0.16^**^ | 1.70^***^ |
|  | **Model 4^1^** | **Model 5^1^** | **Model 6^1^** |
|  | **Self-rated Health** | **Cognitive function** | **CES-D-10 score** |
| **Parenting behaviors** |  |  |  |
| Maternal affection | 0.04^***^ | 0.05 | -0.15^*^ |
| Maternal discipline | -0.01 | -0.03 | 0.15^**^ |
| Paternal affection | 0.05^***^ | 0.10^**^ | -0.29^***^ |
| Paternal discipline | -0.02^*^ | -0.02 | 0.02 |
| Age | -0.01^***^ | -0.07^***^ | 0.04^***^ |
| Male (ref=female) | 0.18^***^ | 0.60^***^ | -1.70^***^ |
| Survey year (ref=2011) |  |  |  |
| 2013 | 0.04^**^ | 0.03 | -0.44^***^ |
| 2015 | 0.05^***^ | -0.16^***^ | -0.36^***^ |
| Rural residence (ref=urban residence) | -0.10^***^ | -0.86^***^ | 1.12^***^ |
| Marital status |  |  |  |
| separated/divorced/widowed | -0.09 | -0.60^**^ | 1.93^***^ |
| single | -0.29^**^ | -1.23^***^ | 2.70^***^ |
| Education attainment score | 0.03^***^ | 1.80^***^ | -0.64^***^ |
| Financial status | 0.01^*^ | 0.12^***^ | -0.01 |
| Childhood socioeconomic disadvantages | -0.06^***^ | -0.17^***^ | 0.50^***^ |
| Childhood serious physical illness (ref=none) | -0.18^***^ | -0.23^*^ | 1.21^***^ |
| Parental mental illness (ref=none) | -0.13^***^ | -0.16^**^ | 1.67^***^ |

^***^P<0.001; ^**^P<0.01; ^*^P<0.05.

Ref; reference.

CES-D-10; 10-item Centre for Epidemiological Studies Depression Scale.
